# Central retinal vein occlusion in a patient using the antipsychotic drug olanzapine: a case report

**DOI:** 10.1186/s13256-021-02865-8

**Published:** 2021-05-29

**Authors:** Ali Nowrouzi, Sepideh Kafiabasabadi, Mario Rodriguez-Calzadilla, Javier Benitez-del-Castillo, Alejandro Soto-Guerrero, Antonio Diaz-Ramos, Kyara Vaneska Marques-Cavalcante

**Affiliations:** 1 Ophthalmology Department of Hospital universitario SAS Jerez de la Frontera, Jerez de la Frontera, Spain; 2 Western University Collage of Dental Medicine, Pamona, California USA; 3 Ophthalmology Department of Hospital general Universitario de Alicante, Alicante, Spain

**Keywords:** Central retinal vein occlusion, Antipsychotic drug, Venous thromboembolism, Macular edema, Olanzapine

## Abstract

**Background:**

We report our findings in a patient who developed central retinal vein occlusion (CRVO) and was a chronic user of olanzapine, an antipsychotic medication.

**Case presentation:**

A 50-year-old Caucasian man, non-smoker, was referred to our clinic with the chief complaint of floater appearance in his left eye for the past 3 days. His past medical history indicated that he had been taking antipsychotic drugs (olanzapine) for about 3 years, with no other systemic disease or risk factors for CRVO.

In the examination, his best-corrected visual acuity (BCVA) was 0.7 in the left eye. The fundus showed signs of nonischemic CRVO with subhyaloid hemorrhage and intraretinal hemorrhage in the posterior pole and superior and inferior retina, without macular edema, confirmed by optical coherence tomography (OCT).

We ruled out other probable differential diagnoses and risk factors which lead to CRVO through a complete physical exam and blood analysis (complete blood count, glucose, urea, creatinine, lipid profile, erythrocyte sedimentation rate, C-reactive protein, prothrombin time, partial thromboplastin time, Bleeding time (BT), fibrinogen level, proteins, antiphospholipid antibodies, homocysteine blood level, antithrombin III, protein C and S, factor V Leiden, prothrombin mutation, angiotensin-converting enzyme level, other autoantibodies, and human leukocyte antigen [HLA]-B51). Finally, we confirmed the probable side effect of olanzapine in CRVO, which has not been previously reported. A possible pro-thrombogenic mechanism of olanzapine at the molecular level is an affinity for 5-HT_2A_serotonin receptors. Blocking these receptors results in increased platelet aggregation and increased blood coagulability.

**Conclusions:**

These results indicate that CRVO can be a complication of chronic use of antipsychotic medications such as olanzapine, as shown for the first time in our case report. Clinicians should question patients who develop a sudden CRVO whether they are using antipsychotic medications such as olanzapine.

## Background

It has been shown that some antipsychotic drugs are risk factors for venous thromboembolic (VTE) diseases such as deep venous thrombosis and pulmonary embolism [1–4]. This has been reported in large primary care populations in previous studies, especially in the Taiwanese population [5, 6]. However, the association between antipsychotic drugs such as quetiapine fumarate, risperidone, and sulpiride (Dogmatyl™) and central retinal vein occlusion (CRVO) has rarely been reported [7].

We present our findings here, for the first time, of CRVO in a patient who had been taking the antipsychotic drug olanzapine for an extended period, after ruling out other probable causes of CRVO in our case.

## Case presentation

A 50-year-old Caucasian man, nonsmoker, was referred to our clinic with the chief complaint of floaters in his left eye for the past 3 days, with no significant family history. He had been diagnosed with bipolar disorder 8 years earlier and was taking olanzapine, fenofibrate, and bisoprolol. He had no other systemic disease or risk factors for CRVO.

**Fig. 1:**
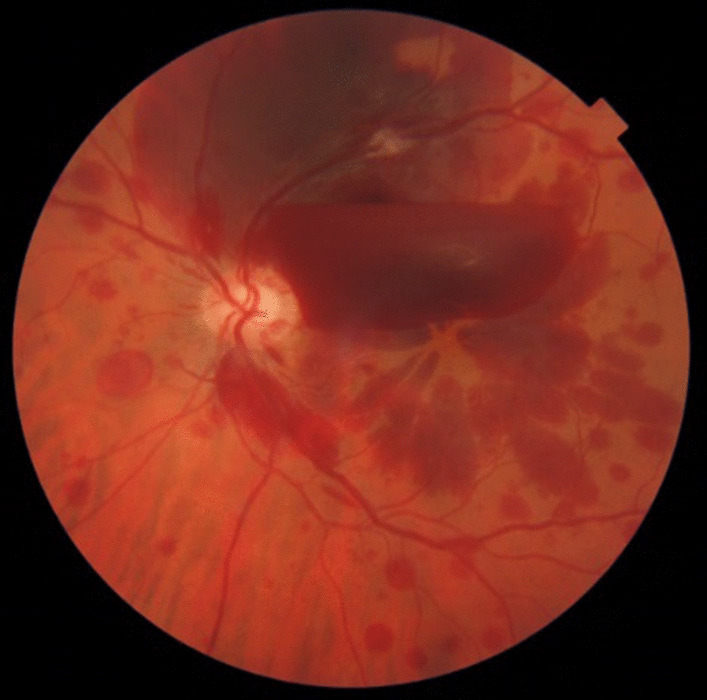
Fundus photograph, signs of central retinal vein occlusion, with tortuous and dilated retinal veins and hemorrhages, with subhyaloid hemorrhage

**Fig.2:**
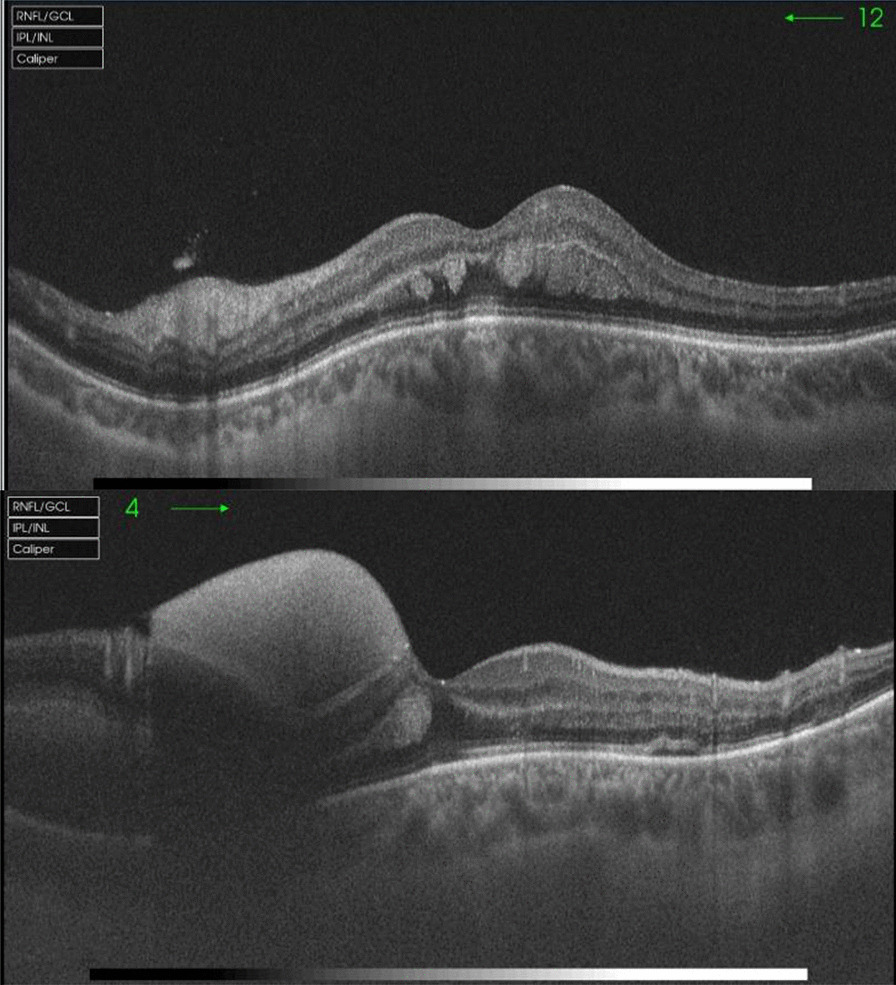
Optical coherence tomography showing subhyaloid hemorrhage and intraretinal hemorrhage in the posterior pole and superior and inferior retina, without macular edema

Upon examination, his best-corrected visual acuity (BCVA) was 0.7 in the left eye and 1 in the right eye, with normal pupillary reflexes and with no relative afferent pupillary defect (RAPD) in either eye. His intraocular pressure (IOP) was 18 mmHg in his right eye and 20 mmHg in his left eye. Slit-lamp examination of the anterior segment of both eyes was normal, without cataracts. The fundus showed signs of CRVO, with tortuous and dilated retinal veins and hemorrhages, with subhyaloid hemorrhage and intraretinal hemorrhage in the posterior pole and superior and inferior retina, without macular edema (Fig.1), confirmed by optical coherence tomography (OCT) (Fig. 2). In the fundus examination, there was no sign of neovascularization elsewhere (NVE) or at the disc level (NVD).

**Fig.3:**
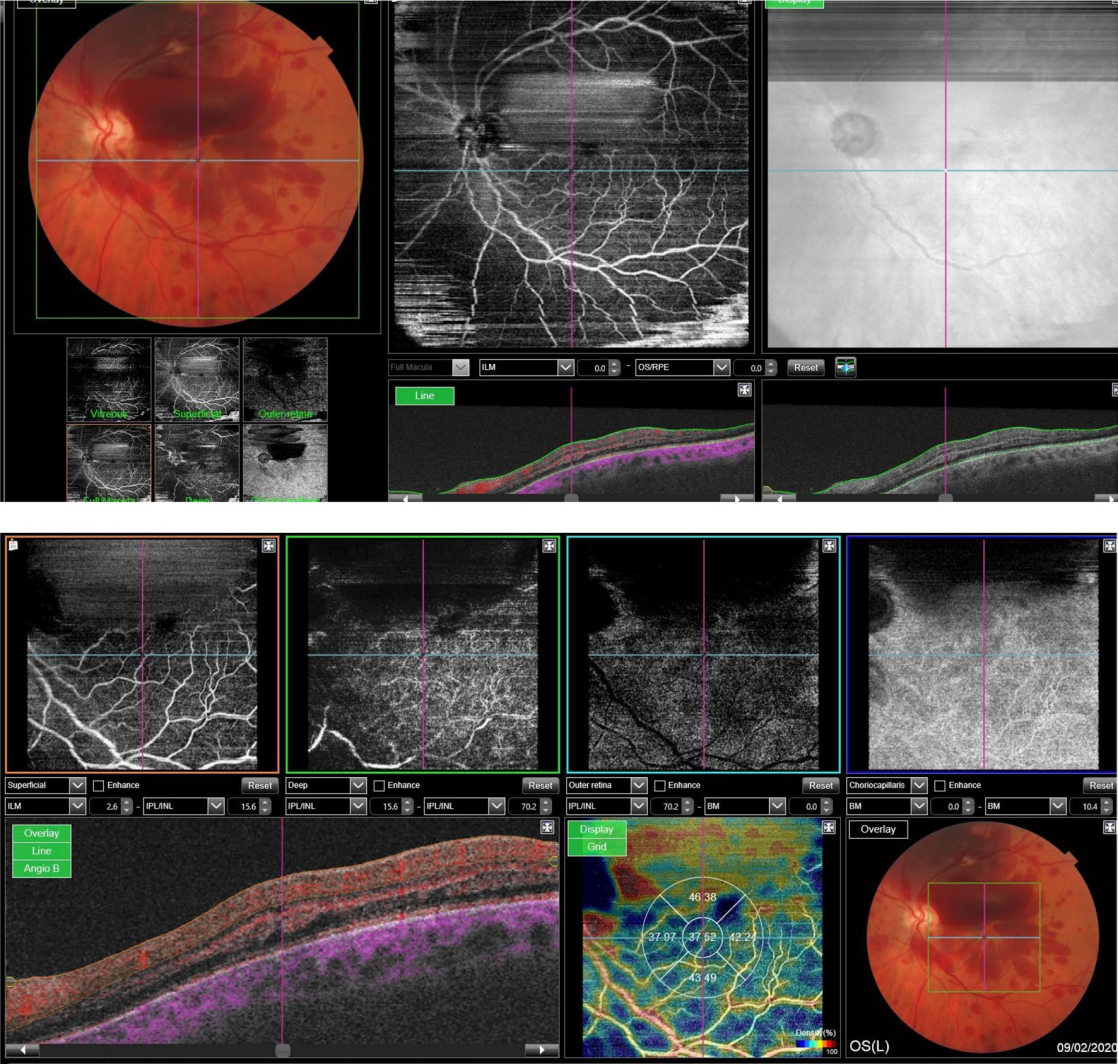
Optical coherence tomography angiography showing subhyaloid hemorrhage and intraretinal hemorrhage without any significant zone of ischemia and neovascularization.

In fluorescein angiography and OCT angiography (Fig. 3), although we could not completely visualize the posterior pole because of the large subhyaloid hemorrhage, there was no significant capillary nonperfusion area or ischemic area, with no posterior segment neovascularization, and we could not detect any leakage to confirm macular edema.

**Fig.4:**
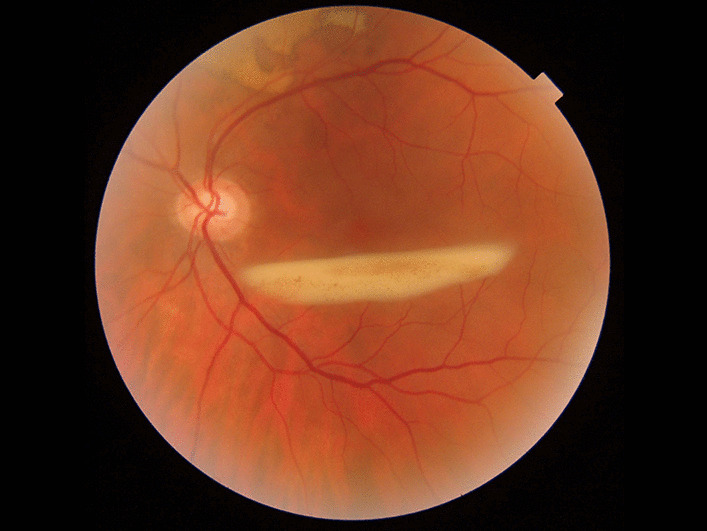
Fundus photograph: exudation of previous subhyaloid hemorrhage and exudation of previous subretinal hemorrhage in the superior juxtapapillary macular edema

Although we could not completely exclude the ischemic classification of thrombosis because of subhyaloid hemorrhage, other findings such as visual acuity and the type of thrombosis seemed to be nonischemic (Fig. 4).

We ruled out other probable differential diagnoses and risk factors which lead to CRVO through complete physical exam including blood pressure control in 24 hours, chest X-ray and whole-body computed tomography scan to identify any other hemorrhage, and blood analysis including complete blood count (CBC), glucose, urea, creatinine, lipid profile, erythrocyte sedimentation rate (ESR), C-reactive protein, prothrombin time (PT), partial thromboplastin time (PTT), bleeding time (BT), fibrinogen level, proteins, antiphospholipid antibodies (APA), homocysteine blood level, antithrombin III, protein C and S, factor V Leiden, prothrombin mutation, angiotensin-converting enzyme (ACE) level, other autoantibodies, and human leukocyte antigen (HLA)-B51.

Finally, we confirmed the probable side effect of olanzapine as another antipsychotic medication in his recent CRVO which was not previously reported.

We discontinued olanzapine to prevent any further complications related to this drug, and we added salicylic acid with an anticoagulant dose of 100 µg per day.

In the second follow-up visit after 2 months, his BCVA was 0.7 in the left eye and 1 in the right eye. His IOP was 16 mmHg in his right eye and 18 mmHg in his left eye.

Gonioscopy was performed by Goldmann gonioscopy, and the presence of neovascularization in the angle was ruled out. His fundus showed signs of exudation of previous subhyaloid and subretinal hemorrhage in the superior juxtapapillary zone, without any NVE.

## Discussion

The pathogenesis of CRVO has not been definitively determined [8]. The most commonly recognized risk factors are age and systemic vascular disorders, although rarely patients under the age of 45 can develop CRVO [9]. Some cases of CRVO are associated with thrombophilia and hematologic disorders [10, 11], and CRVO can occur due to a combination of different systemic disorders, such as hemodynamic changes (venous stasis), degenerative changes in the blood vessel walls, and blood hypercoagulability states. It has also been suggested that hyperviscosity due to high hematocrit levels or other causes may play a role in the development of CRVO. The higher blood viscosity increases the aggregation of erythrocytes and slows the blood flow, thus increasing the risk of clotting [10, 11].

A primary risk factor for the development of CRVO is age, with 90% of patients older than 50 years. Systemic arterial hypertension, open-angle glaucoma, diabetes mellitus, and hyperlipidemia have all been implicated as other primary risk factors for CRVO. Other associated risk factors include smoking, optic disc drusen, optic disc edema, hypercoagulable state (polycythemia, multiple myeloma, cryoglobulinemia, Waldenström macroglobulinemia, antiphospholipid syndrome, Leiden factor V, activated protein C resistance, hyperhomocysteinemia, protein C and S deficiency, antithrombin III mutation, prothrombin mutation), syphilis, sarcoidosis, African American race, sickle cell disease, human immunodeficiency virus (HIV), vasculitis, drugs such as oral contraceptives or diuretics, abnormal platelet function, orbital disease, and rarely migraines [12].

Severe thromboembolic diseases such as pulmonary embolism and VTE have been reported to develop in patients being treated with antipsychotic agents, but the incidence was found to be as low as 0.0091% [2]. Antipsychotic drugs contribute to the development of a hypercoagulable state through several potential mechanisms, including drug-induced sedation, obesity, hyperprolactinemia, increased platelet aggregation, and elevated APA levels [13, 14].

A search of PubMed with the keywords “central retinal vein occlusion and antipsychotic drug” and “branch retinal vein occlusion and antipsychotic drug” yielded only three publications, by Taki *et al.* [7], Agca *et al.* [15], and Yong *et al.* [16].

Metabolic symptoms caused by olanzapine represent an indirect mechanism for VTE development. The pro-thrombogenic metabolic symptoms that often occur during olanzapine treatment include hyperglycemia, hyperleptinemia, dyslipidemia, and weight gain [17]. In our case, we ruled out these indirect effects of olanzapine by the patient’s lab test results showing normoglycemia, no hyperleptinemia, and normal lipid profile. The patient's body mass index (BMI) was 25, classified as overweight, but obesity was not a clear risk factor in our case.

Our case report is the first to report the use of olanzapine associated with CRVO, which we were able to confirm as the probable side effect of this drug. Although olanzapine side effects were previously reported as a precipitating factor for VTE formation [18], CRVO as a probable side effect of this drug has not been published previously.

A possible pro-thrombogenic mechanism of olanzapine at the molecular level is an affinity for 5-HT_2A_serotonin receptors. Blocking these receptors results in increased platelet aggregation and increased blood coagulability [19]. Blockade of α_1_ adrenergic receptors by olanzapine may cause hypotension and therefore venous stasis in the lower limbs. Another mechanism may be olanzapine-induced production of the APAs lupus anticoagulant (LA) and anticardiolipin antibodies (ACLAs) [20]. Increased APA titers is associated with a pro-thrombogenic state [17]. We ruled out increased LA and ACLAs by normal lab test results in our case report. Olanzapine may also induce a temporary increase in prolactin levels early in the course of treatment. Hyperprolactinemia correlates with increased levels of P-selectin, a platelet activation marker [21].

Olanzapine has a high affinity for 5-HT_2A_, a serotonin receptor. 5-HT_2A_ blockers stimulate 5-HT_2A_ in the platelets and induce platelet aggregation, causing the vascular smooth muscles to contract [22, 23]. Thus, platelet aggregation may have played a role in CRVO in our patient.

This study does have limitations. The patient took other drugs, and we did not determine which was the real culprit, although there is no other drug in his past medical history that has any kind of confirmed thrombophilia. It would be helpful to measure the retinal venous pressure, particularly in the unaffected contralateral eye [24, 25]. If the retinal venous pressure is dependent on the intake of these antipsychotic drugs, we would have better evidence of a relationship between CRVO and antipsychotic drug use.

## Conclusions

These results indicate that CRVO can be a complication of chronic use of antipsychotic medications such as olanzapine, as shown here for the first time in our case report. Clinicians should question patients who develop a sudden CRVO whether they are using antipsychotic medications such as olanzapine.

## Data Availability

All supporting data are available to be sent to the editorial department when it is necessary.

## Abbreviations

CRVO: Central retinal vein occlusion
OCT: Optical coherence tomography
BCVA: Best-corrected visual acuity
VTE: Venous thromboembolic
